# The Secretaryship of St. Mark's

**Published:** 1914-08-08

**Authors:** 


*To the Editor of* The Hospital.

### The Secretaryship of St. Mark's.

*To the Editor of* The Hospital.

Sir,—Referring to Mr. Lockhart Mummery's letter in your last issue, I should like to mention that he is not correct as to his statement that the emoluments could be ascertained by inquiring at the hospital.

My assistant applied, and was selected to appear before the Selection Committee; but in no way could he find out the salary offered, neither from the hospital nor did the committee inform him.

I enclose my card and beg to sign myself, Yours faithfully,

"Hospital Officer."

Primrose Club, Park Place, St. James's.

[This letter is typical of others at our disposal which show the feeling that is certainly prevalent in relation to this appointment. In spite of Mr. Lockhart Mummery's disclaimer, it is asserted that the appointment was not advertised in the ordinary manner.—Ed., The Hospital.]

